# Adult non-invasive pneumococcal pneumonia in Portugal is dominated by serotype 3 and non-PCV13 serotypes 3-years after near universal PCV13 use in children

**DOI:** 10.3389/fpubh.2023.1279656

**Published:** 2023-12-20

**Authors:** Catarina Silva-Costa, Joana Gomes-Silva, Andreia Santos, Mário Ramirez, José Melo-Cristino, The Portuguese Group for the Study of Streptococcal Infections

**Affiliations:** Instituto de Microbiologia, Instituto de Medicina Molecular, Faculdade de Medicina, Universidade de Lisboa, Lisbon, Portugal

**Keywords:** pneumonia, serotypes, vaccines, antimicrobial resistance (AMR), *Streptococcus pneumoniae* (pneumococcus), epidemiology

## Abstract

**Introduction:**

Non-invasive pneumococcal pneumonia (NIPP) is possibly the most frequent infection by *Streptococcus pneumoniae* in adults. However, the herd effect of vaccinating children in adult NIPP (aNIPP) remains poorly characterized.

**Methods:**

We determined the serotype distribution and antimicrobial susceptibility of isolates causing aNIPP (>18 years) in 2016–2018 in Portugal; 3 years with near universal vaccination of children with the 13-valent conjugate vaccine (PCV13), following over a decade of significant PCV use in children in the private market.

**Results and discussion:**

Among the 1,149 aNIPP isolates, the most frequent serotypes detected were: 3 (*n* = 168, 14.6%), 11A (*n* = 102, 8.9%), 19F (*n* = 70, 6.1%), 23A and 23B (*n* = 62, 5.4% each), 9N (*n* = 60, 5.2%), 8 and 29/35B (*n* = 43, 3.7% each); together accounting for 53% of all isolates. The serotype distribution causing aNIPP was stable in 2016–2018, with the serotypes included in PCV7 still being important causes of disease and serotype 3, a PCV13 serotype, remaining the leading cause of aNIPP. There was an increase in penicillin non-susceptibility from 17% in 2016 to 24% in 2018 (*p* = 0.018). Some PCV13 serotypes, such as 14, 19A and 19F were associated to resistance, which may have contributed to their persistence. The fact that close to 20% of aNIPP is caused by four non-vaccine serotypes (23A, 23B, 9N, and 29/35B) and that there were significant differences in serotype distribution relative to invasive disease, stress the importance of maintaining the surveillance of these infections. The lack of a continued herd effect from vaccinating children and the significant fraction of aNIPP potentially preventable by PCV13 (30%), PCV15 (34%), PCV20 (53%) and the 23-valent polysaccharide vaccine (61%) underscore the importance of considering the broader use of pneumococcal vaccines in adults.

## Introduction

*Streptococcus pneumoniae* is an important human pathogen and the most frequent cause of community acquired pneumonia in adults (1). Pneumococcal pneumonia can be invasive (bacteremic) or non-invasive (non-bacteremic) and, although the latter is more frequent than bacteremic pneumonia, less is known regarding the epidemiology of non-invasive pneumococcal pneumonia (NIPP) (2). Worldwide, the use of pneumococcal conjugate vaccines (PCVs) in children led to decreases in the incidence of pediatric invasive pneumococcal disease (IPD), but also of IPD among non-vaccinated adults (3, 4). This was driven mostly by decreases of PCV serotypes in adult IPD (aIPD), as was also seen in Portugal (5–7). Higher valency conjugate vaccines – PCV15 and PCV20 (8, 9) – are entering the market for use in adults, potentially offering additional benefits for the direct prevention of pneumococcal disease in adults and other conjugate vaccines – PCV21 and PCV24 – are already in clinical trials (10, 11). In contrast to well documented effect of children vaccination in the serotypes and incidence of aIPD its effect in adult NIPP (aNIPP) is less clear (12).

Worldwide, some non-PCV serotypes emerged as important causes of pneumococcal disease (4, 5, 7, 12, 13) following PCV use. It is becoming increasingly evident that the serotype distributions in IPD and NIPP are different and it is unclear if the changes seen in serotypes causing aIPD can be directly extrapolated for aNIPP (5, 14, 15). Given this uncertainty, knowing the serotype distribution in aNIPP is critical, not only to evaluate potential changes due to children vaccination with PCVs but also the potential benefits of direct PCV use in adults.

Both the 7-valent and the 13-valent PCV (PCV7 and PCV13) have been used in children in Portugal since becoming available, with the later having been introduced in the National Immunization Program in 2015 and quickly reaching an uptake >95%. The 23-valent polysaccharide vaccine (PPV23) has also been available for adults and, since 2015, the sequential vaccination with PCV13 and PPV23 is recommended by the national health authorities for specific adult risk-groups (16). Although recommendations for their use in all adults aged ≥65 years were made by two medical societies (17, 18), the uptake of pneumococcal vaccines in adults is Portugal is estimated to be low. Possibly because of vaccine use in children, in 2010–2015 the proportion of PCV13 serotype aNIPP declined, mainly due to decreases in importance of serotypes 3 and 19A, while non-vaccine serotypes (NVTs, serotypes not included in any pneumococcal vaccine currently available) increased (5, 19). Moreover, a comparison between the serotypes causing aIPD and aNIPP revealed that some serotypes were specifically associated with each of these disease presentations (5, 19). On the other hand, in 2015–2018 PCV13 serotypes still persisted as causes of aIPD, especially serotypes 3, 14 and 19A, and there was a marked increase of non-PCV serotype 8 (7).

In this study we determined serotype distribution in aNIPP in 2016–2018, to evaluate the potential impact of the increased uptake of PCV13 in children since its introduction in the National Immunization Plan in 2015. Isolates were characterized for serotype and antimicrobial susceptibility and the results were compared with previous aNIPP data and contemporary aIPD data to evaluate trends and the propensity of serotypes to cause each of these disease presentations.

## Materials and methods

### Bacterial isolates

Isolates were provided by a laboratory-based surveillance system that includes 30 microbiology laboratories throughout Portugal (Supplementary Figure S1). Our surveillance system is approved by the CAML institutional review board (258/22). The participating laboratories were asked to submit all consecutively collected pneumococci causing infections to the central laboratory. Although the laboratories were contacted periodically to submit the isolates to the central laboratory, no audit was performed to ensure compliance, which may be variable in this type of study. The identification of all isolates as *Streptococcus pneumoniae* was confirmed by colony morphology and hemolysis on blood agar plates, optochin susceptibility and bile solubility. The isolates included in this study were recovered from sputum, bronchial secretions or bronchoalveolar lavage of adult patients (≥18 years) with a presumptive diagnosis of pneumonia, between 2016 and 2018. Isolates were excluded when pneumococci were simultaneously isolated from blood or another usually sterile product, and when other potential bacterial pathogens besides pneumococci were detected in the sample (such as *Haemophilus influenzae*, which was also frequently detected). Only one isolate from each episode was considered. All participating laboratories determine the quality of sputum samples according to international guidelines, and only high-quality samples were considered for further testing.

### Serotyping and antimicrobial susceptibility testing

Serotyping was performed by the standard capsular reaction test using the chessboard system and specific sera (Statens Serum Institut, Copenhagen, Denmark) (20). Serotypes were classified into vaccine serotypes, i.e., those included in PCV7 (serotypes 4, 6B, 9V, 14, 18C, 19F, 23F), in PCV13 (all PCV7 serotypes and the additional serotypes present only in PCV13, addPCV13: 1, 3, 5, 6A, 7F, and 19A), in PPV23 (all PCV13 serotypes, except serotype 6A, and the additional serotypes present only in PPV23, addPPV23: 2, 8, 9N, 10A, 11A, 12F, 15B, 17F, 20, 22F, and 33F) and non-vaccine serotypes (NVT, including all other serotypes). We also defined the PCV15 serotypes (all PCV13 serotypes plus serotypes 22F and 33F), and PCV20 serotypes (all PCV15 serotypes and serotypes: 8, 10A, 11A, 12F, and 15B/C), both subsets of serotypes included in PPV23. Given the high frequency of spontaneous switching between serotypes 15B and 15C we opted to group isolates with these serotypes into a single group and consider it included in PCV20 and PPV23 (although only serotype 15B is included in these vaccines). Due to difficulties in phenotypically distinguishing isolates of serotype 25A and serogroup 38 and of serogroup 29 and serotype 35B, these were also grouped together into the 25A/38 and 29/35B groups. The isolates that were not typable with any of the complete set of sera were considered non-typable (NT). Minimum inhibitory concentrations (MICs) for penicillin and cefotaxime were determined using Etest strips (Biomérieux, Marcy-L'Etoile, France). The results were interpreted using the Clinical and Laboratory Standards Institute (CLSI) recommended breakpoints (21). In the case of penicillin, unless otherwise stated, the breakpoints for oral penicillin V were used, allowing the comparison with previously published data on penicillin resistance. The Kirby–Bauer disk diffusion assay was used to determine susceptibility to levofloxacin, erythromycin, clindamycin, chloramphenicol, trimethoprim/sulfamethoxazole, tetracycline, vancomycin and linezolid, according to the CLSI recommendations and interpretative criteria (22). Macrolide resistance phenotypes were identified using a double disc test with erythromycin and clindamycin, as previously described (23). The MLS_B_ phenotype was defined as the simultaneous resistance to erythromycin and clindamycin, while the M phenotype (resistance to macrolides) was defined as non-susceptibility only to erythromycin. Differences were evaluated by the Fisher exact test, and the Cochran-Armitage test (CA) was used for trends with the false discovery rate (FDR) correction for multiple testing (24). A *p* < 0.05 was considered significant for all tests.

## Results

### Serotype distribution

A total of 1,149 isolates from aNIPP were collected: 389 in 2016, 389 in 2017 and 371 in 2018. When stratified by age group, 246 isolates (21.4%) were recovered from patients 18–49 years old, 322 isolates (28.0%) from patients 50–64 years old and 581 isolates (50.6%) from patients ≥65 years old. Most isolates were recovered from sputum (*n* = 715, 62.2%), 337 (29.3%) were recovered from bronchial secretions and 97 (8.4%) from bronchoalveolar lavage fluid. A total of 51 different serotypes were detected with the most frequent serotypes being: 3 (*n* = 168, 14.6%), 11A (*n* = 102, 8.9%), 19F (*n* = 70, 6.1%), 23A and 23B (*n* = 62, 5.4% each), 9N (*n* = 60, 5.2%), 8 and 29/35B (*n* = 43, 3.7% each), together accounting for 53% of all isolates. 24 isolates (2.1%) were non-typeable (NT).

**Figure 1:**
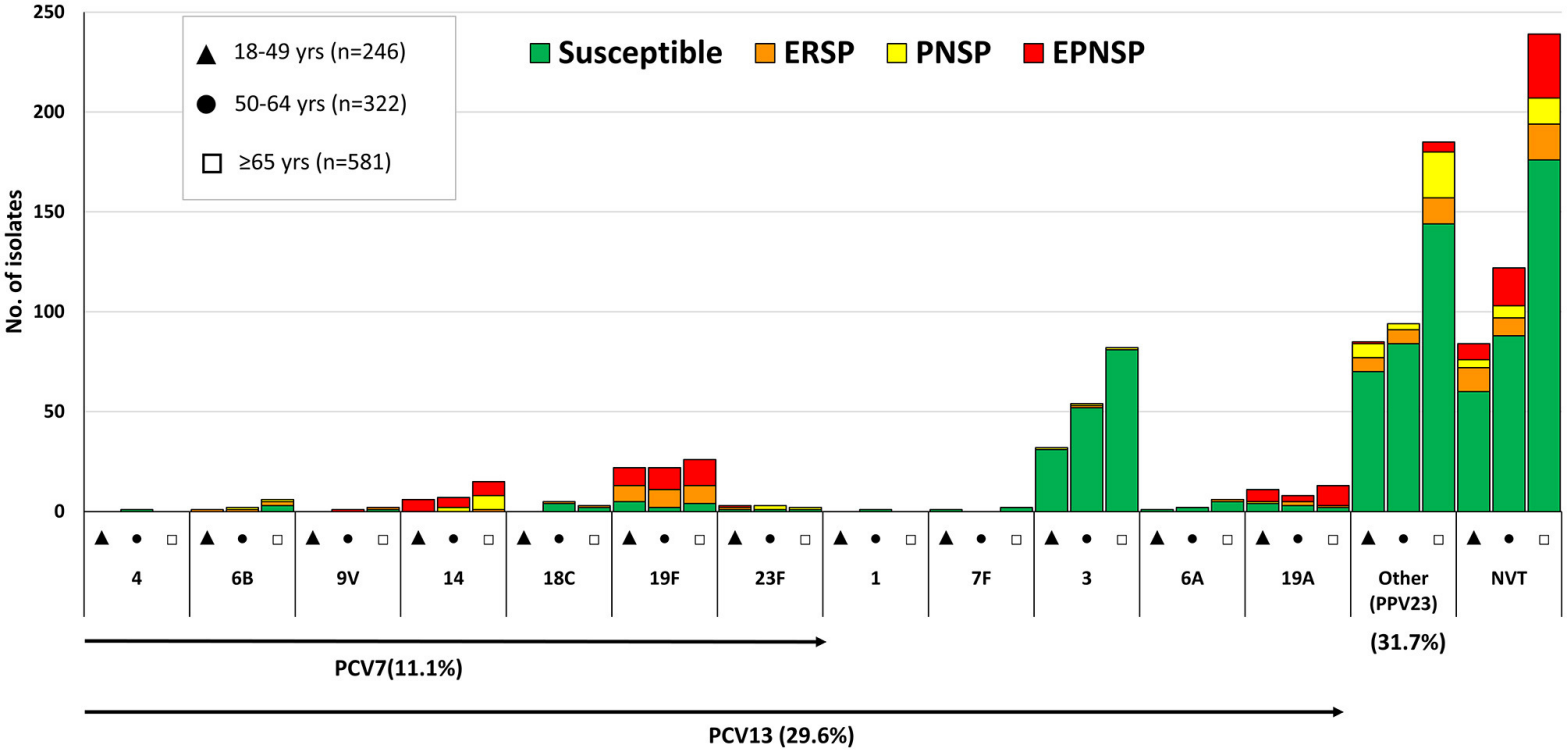
Serotypes of isolates causing non-invasive pneumococcal pneumonia in adult patients (≥18 years) in Portugal, 2016–2018. The number of isolates expressing each serotype in each of the age groups considered is indicated. Isolates recovered from patients 18–49 years are indicated by black triangles, from patients 50–64 years by black circles, and from patients ≥65 years by open squares. Isolates presenting both erythromycin resistance and penicillin non-susceptibility (EPNSP) are represented by red bars. Penicillin non-susceptible isolates (PNSP) are indicated by yellow bars. Erythromycin resistant pneumococci (ERP) are indicated by orange bars. Isolates susceptible to both penicillin and erythromycin are represented by green bars. The serotypes included in the 7-valent conjugate vaccine (PCV7) and in the 13-valent conjugate vaccine (PCV13) are indicated by the arrows. Serotype 5 was not found in our collection. NVT, non-vaccine serotypes. Other (PPV23), the additional serotypes included in the 23-valent polysaccharide vaccine and not present in PCV13.

**Figure 2:**
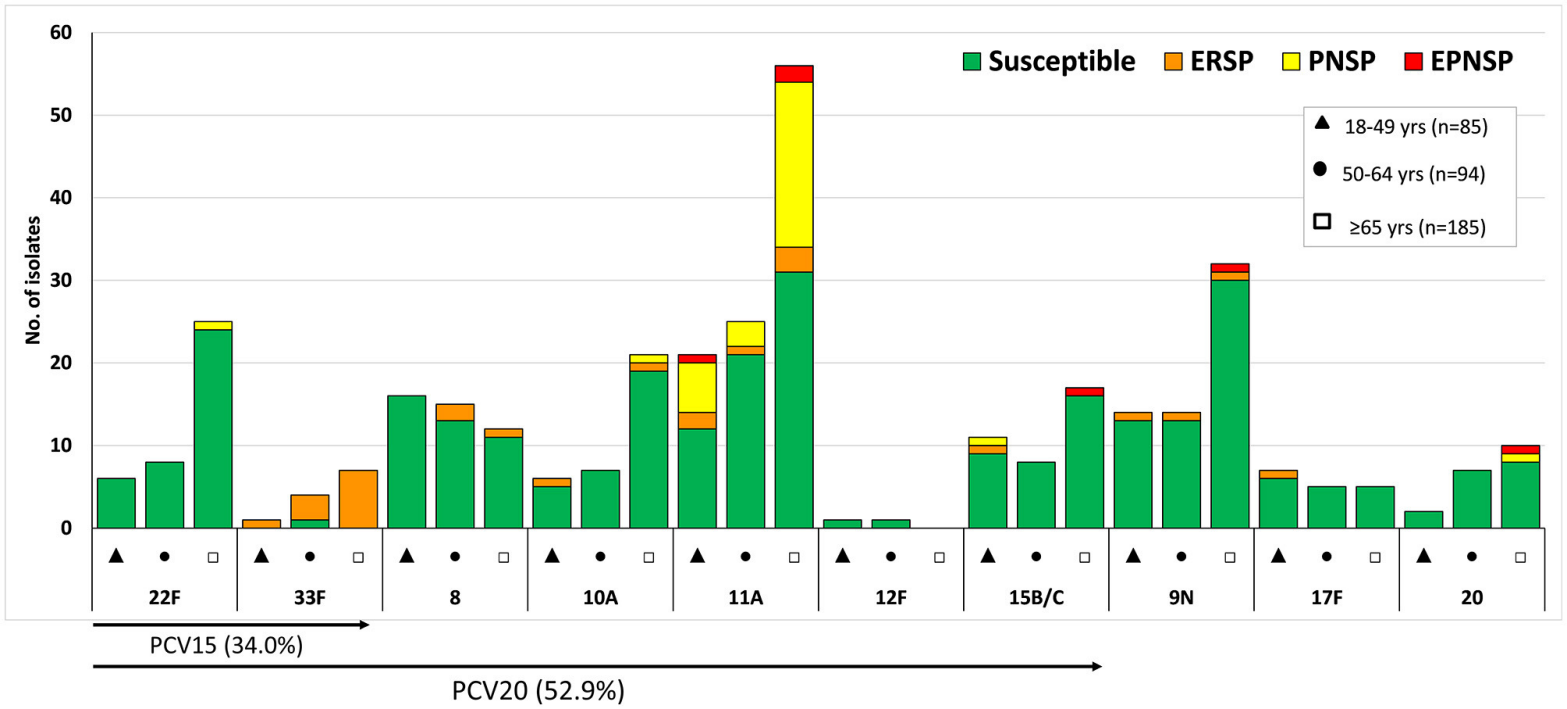
Isolates expressing serotypes present in PPV23 but not included in PCV13 causing non-invasive pneumococcal pneumonia in adult patients (≥18 years) in Portugal, 2016–2018. See legend of Figure 1. Out of the 11 serotypes present in PPV23 but absent from PCV13, serotype 2 was not found in our collection. The serotypes exclusively included in the 15-valent conjugate vaccine (PCV15) and in the 20-valent conjugate vaccine (PCV20) are indicated by the arrows, but the proportions indicated corresponds to all the serotypes included in each PCV.

**Figure 3:**
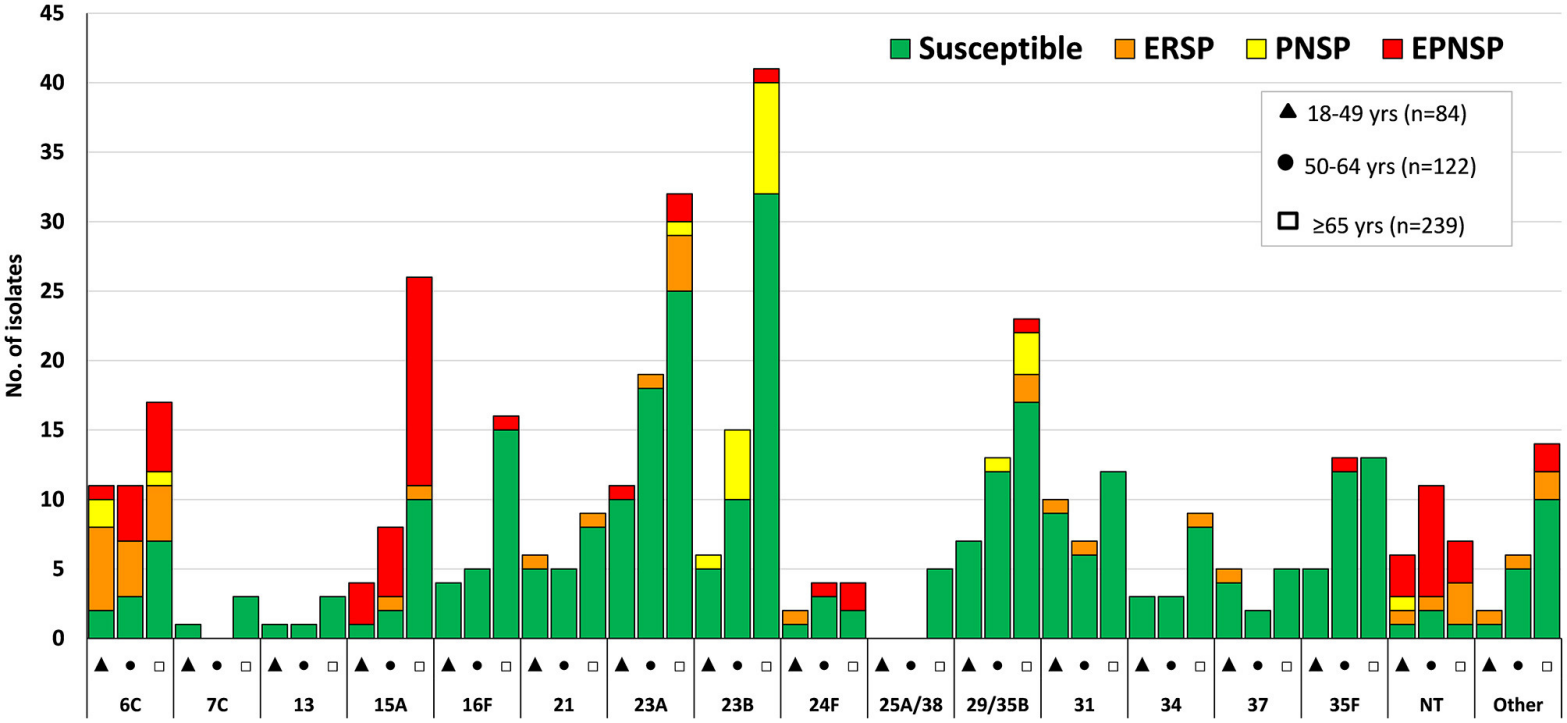
Isolates expressing serotypes not included in any pneumococcal vaccine causing non-invasive pneumococcal pneumonia in adult patients (≥18years) in Portugal, 2016–2018. See legend of Figure 1. NT, non-typable. Isolates expressing serotype 25A and 38, and 29 and 35B could not be distinguished phenotypically and are represented together. Only serotypes including n> 3 isolates are discriminated. Other include serotypes 6D and 35A (*n* = 3 each); 7B, 11D and 18A (*n* = 2 each); 10B, 11C, 11F, 12B, 18F, 19B, 33A, 36 and 47F (*n* = 1 each).

Overall, a significant proportion of isolates still expressed serotypes included in PCV7 (11.1%, *n* = 127), while 29.6% of the isolates (*n* = 340) expressed serotypes included in PCV13 (Figure 1). As for the newer conjugate vaccines, only 4.4% (*n* = 51) of the isolates expressed the additional serotypes included in PCV15 (22F and 33F), while 18.9% of the isolates (*n* = 217) expressed the additional PCV20 serotypes (8, 10A, 11A, 12F, and 15B/C) (Figure 2). The isolates expressing the addPPV23 serotypes (31.7%, *n* = 364, Figure 2) and NVT serotypes (38.7%, *n* = 445, Figure 3) accounted for similar proportions of aNIPP. Four NVTs (23A, 23B, 9N, and 29/35B) accounted for almost a fifth (19.8%) of all aNIPP cases.

The distribution of serotypes among the age groups is shown in Figures 1–3 and in Table 1 for serotypes expressed by >10 isolates. Serotypes 23B and 15A were associated with older adults (≥65 years old, *p* = 0.001 and *p* = 0.024, respectively, but only the first was supported after FDR correction), while the opposite was observed for serotypes 8, 17F and 19F (*p* = 0.001, *p* = 0.033 and *p* = 0.011, respectively, again only the first supported after FDR correction). When considering the serotypes included in each of the current and future pneumococcal vaccines, no significant differences, after FDR correction, were seen when comparing the different age groups (Table 1).

**Table 1:** Serotype distribution in each age group (*n* > 10 isolates) in Portugal, 2016–2018.

**Serotype**	**No of isolates (%)**			**CA^a^**
	**(18, 49) years**	**(50, 64) years**	≥**65 years**	
3	32 (13.0)	54 (16.8)	82 (14.1)	0.915
11A	21 (8.5)	25 (7.8)	56 (9.6)	0.493
19F	22 (8.9)	22 (6.8)	26 (4.5)	0.011
23A	11 (4.5)	19 (5.9)	32 (5.5)	0.632
23B	6 (2.4)	15 (4.7)	41 (7.1)	**0.001**
9N	14 (5.7)	14 (4.3)	32 (5.5)	0.933
8	16 (6.5)	15 (4.7)	12 (2.1)	**0.001**
29/35B	7 (2.8)	13 (4.0)	23 (4.0)	0.499
6C	11 (4.5)	11 (3.4)	17 (2.9)	0.272
22F	6 (2.4)	8 (2.5)	25 (4.3)	0.119
15A	4 (1.6)	8 (2.5)	26 (4.5)	0.024
15B/C	11 (4.5)	8 (2.5)	17 (2.9)	0.339
10A	6 (2.4)	7 (2.2)	21 (3.6)	0.266
19A	11 (4.5)	8 (2.5)	13 (2.2)	0.099
35F	5 (2.0)	13 (4.0)	13 (2.2)	0.812
31	10 (4.1)	7 (2.2)	12 (2.1)	0.128
14	6 (2.4)	7 (2.2)	15 (2.6)	0.841
16F	4 (1.6)	5 (1.6)	16 (2.8)	0.232
NT	6 (2.4)	11 (3.4)	7 (1.2)	0.121
20	2 (0.8)	7 (2.2)	10 (1.7)	0.475
21	6 (2.4)	5 (1.6)	9 (1.5)	0.461
17F	7 (2.8)	5 (1.6)	5 (0.9)	0.033
34	3 (1.2)	3 (0.9)	9 (1.5)	0.596
33F	1 (0.4)	4 (1.2)	7 (1.2)	0.362
37	5 (2.0)	2 (0.6)	5 (0.9)	0.203

^a^CA, Cochran Armitage test for trend. In bold are the significant p-values after FDR correction.

**Figure 4:**
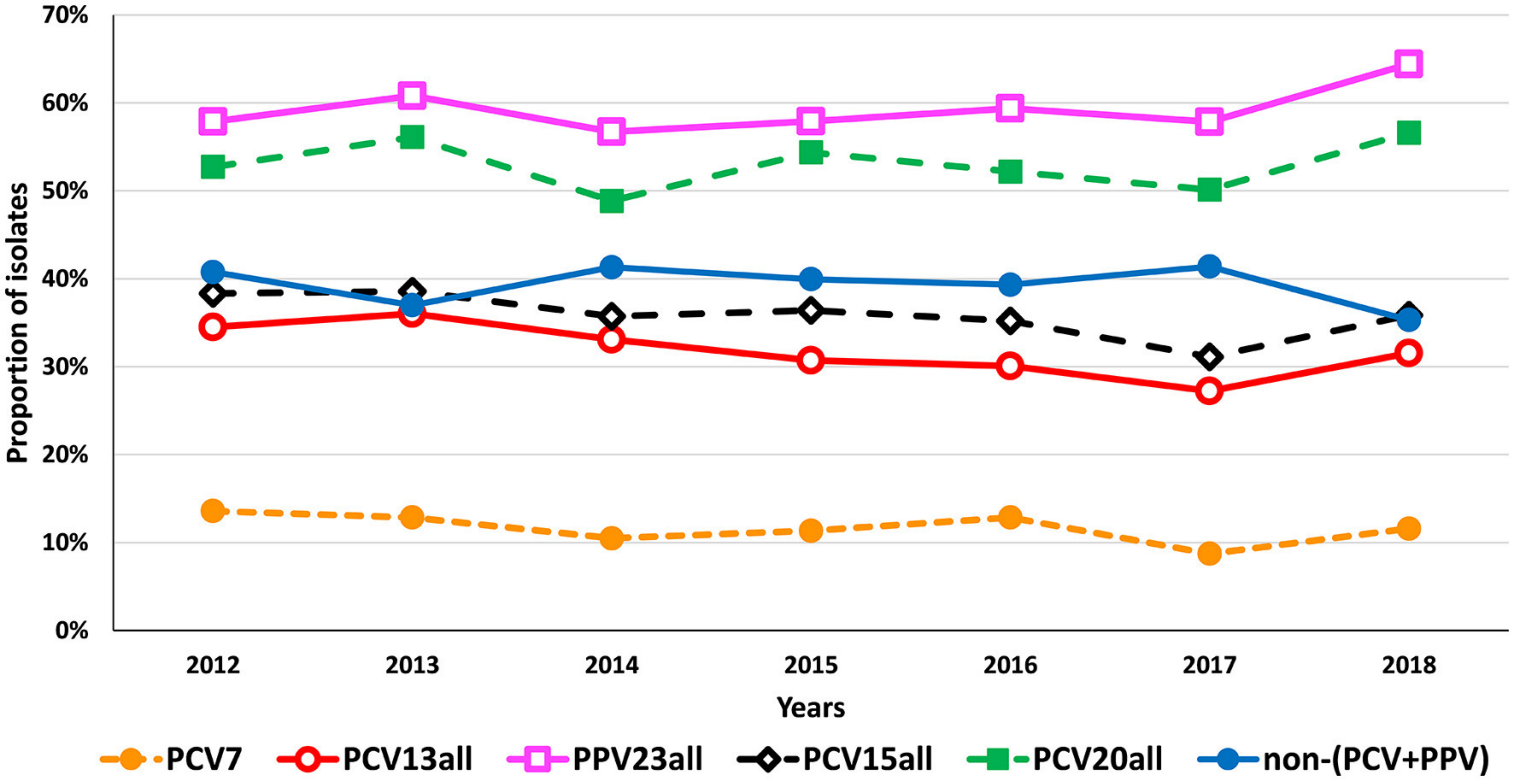
Proportion of isolates expressing the serotypes included in existing and currently entering the market pneumococcal vaccines causing non-invasive pneumococcal pneumonia in adult patients (≥18 years) in Portugal, 2012–2018. The data up to 2015 were presented previously (5).

Overall, in 2016–2018 there were only modest variations in the proportion of aNIPP caused by vaccine serotypes, with rebounds of varying magnitude in the importance of PCV serotype aNIPP in 2018 (Figure 4 and Table 2). When analyzing the whole 2012–2018 period according to the serotype groups defined by the vaccines, it was possible to detect an increase in the proportion of aNIPP caused by the additional serotypes included in PCV20 but not present in PCV13, from 14.4% in 2012 to 20.8% in 2018 (*p* = 0.014, significant after FDR correction), which is apparent in the overall proportion of aNIPP caused by PCV20 serotypes (Figure 4). In the same period, the proportion of isolates expressing the additional serotypes present in PCV10 relative to PCV7 (1, 5, and 7F) decreased from 1.9% in 2012 to 0.5% in 2018 (*p* = 0.011, significant after FDR correction). Changes in individual serotypes in aNIPP from 2012 to 2018 are shown in Table 3, for serotypes detected in ≥3 isolates in at least 1 year. When considering the current study period (2016–2018), decreases were detected in NVTs 35F (*p* = 0.002), and 16F (*p* = 0.008), and an increase in serotype 24F (*p* = 0.044), all not supported after FDR correction. When considering 2012–2018, there was a decrease in the proportion of aNIPP caused by serotype 6A (*p* = 0.041) and 7F (*p* = 0.021) and increases in the proportion of NIPP caused by serotypes 8 (*p* = 0.039) and 23B (*p* = 0.025), but again none were supported after FDR correction.

**Table 2:** Isolates expressing serotypes included in existing and upcoming pneumococcal vaccines responsible for non-invasive pneumococcal pneumonia in adult patients (≥18 years) in Portugal, 2016–2018.

**Vaccine**	**No of isolates (%)**		
	**(18, 49) years**	**(50, 64) years**	≥**65 years**
PCV13	77 (31.3)	106 (32.9)	157 (27)
PCV15	84 (34.1)	118 (36.6)	189 (32.5)
PCV20	139 (56.5)	174 (54)	295 (50.8)
PPV23	161 (65.4)	198 (61.5)	336 (57.8)
PCV21	174 (70.7)	229 (71.1)	442 (76.1)
PCV24	162 (65.9)	200 (62.1)	342 (58.9)

**Table 3:** Serotypes of the isolates responsible for non-invasive pneumococcal pneumonia in adult patients (≥18 years) in Portugal, 2012–2018.

**Serotype^a^**	**No of isolates (%)**							**CA 16–18^b^**	**CA 12–18^b^**
					**Current study period**				
	**2012**	**2013**	**2014**	**2015**	**2016**	**2017**	**2018**		
**PCV13**									
3	48 (13.0)	54 (16.9)	41 (13.4)	53 (12.5)	52 (13.4)	56 (14.4)	60 (16.2)	0.275	0.610
4	1 (0.3)	1 (0.3)	2 (0.7)	3 (0.7)	0 (0.0)	0 (0.0)	1 (0.3)	0.211	0.400
6A	5 (1.4)	7 (2.2)	6 (2.0)	9 (2.1)	5 (1.3)	3 (0.8)	1 (0.3)	0.112	**0.041**
6B	7 (1.9)	4 (1.3)	4 (1.3)	6 (1.4)	3 (0.8)	3 (0.8)	3 (0.8)	0.954	0.102
7F	5 (1.4)	4 (1.3)	4 (1.3)	5 (1.2)	0 (0.0)	1 (0.3)	2 (0.5)	0.146	**0.021**
9V	0 (0.0)	5 (1.6)	0 (0.0)	0 (0.0)	1 (0.3)	1 (0.3)	1 (0.3)	0.973	0.400
14	12 (3.3)	6 (1.9)	6 (2.0)	10 (2.4)	13 (3.3)	8 (2.1)	7 (1.9)	0.191	0.534
18C	4 (1.1)	1 (0.3)	1 (0.3)	1 (0.2)	3 (0.8)	4 (1.0)	1 (0.3)	0.413	0.759
19A	17 (4.6)	9 (2.8)	18 (5.9)	14 (3.3)	10 (2.6)	11 (2.8)	11 (3.0)	0.741	0.104
19F	22 (6.0)	22 (6.9)	15 (4.9)	26 (6.1)	26 (6.7)	16 (4.1)	28 (7.5)	0.639	0.903
23F	4 (1.1)	2 (0.6)	4 (1.3)	2 (0.5)	4 (1.0)	2 (0.5)	2 (0.5)	0.413	0.378
**addPCV15**									
22F	14 (3.8)	8 (2.5)	6 (2.0)	21 (5.0)	15 (3.9)	11 (2.8)	13 (3.5)	0.781	0.827
33F	6 (1.6)	1 (0.3)	7 (2.3)	3 (0.7)	5 (1.3)	4 (1.0)	3 (0.8)	0.518	0.474
**addPCV20**									
11A	29 (7.9)	29 (9.1)	22 (7.2)	40 (9.5)	34 (8.7)	28 (7.2)	40 (10.8)	0.332	0.421
8	7 (1.9)	10 (3.1)	6 (2.0)	16 (3.8)	11 (2.8)	16 (4.1)	16 (4.3)	0.278	**0.039**
15B/C	6 (1.6)	11 (3.4)	7 (2.3)	10 (2.4)	7 (1.8)	16 (4.1)	13 (3.5)	0.172	0.134
10A	11 (3.0)	6 (1.9)	5 (1.6)	10 (2.4)	13 (3.3)	13 (3.3)	8 (2.2)	0.339	0.661
**addPPV23**									
9N	11 (3.0)	13 (4.1)	16 (5.2)	12 (2.8)	20 (5.1)	23 (5.9)	17 (4.6)	0.737	0.122
20	5 (1.4)	5 (1.6)	6 (2.0)	8 (1.9)	9 (2.3)	4 (1.0)	6 (1.6)	0.442	0.994
17F	8 (2.2)	4 (1.3)	8 (2.6)	4 (0.9)	4 (1.0)	6 (1.5)	7 (1.9)	0.314	0.595
**NVT**									
23B	15 (4.1)	8 (2.5)	15 (4.9)	18 (4.3)	16 (4.1)	20 (5.1)	26 (7.0)	0.078	**0.025**
23A	24 (6.5)	12 (3.8)	14 (4.6)	17 (4.0)	27 (6.9)	19 (4.9)	16 (4.3)	0.107	0.692
29/35B	12 (3.3)	6 (1.9)	6 (2.0)	10 (2.4)	9 (2.3)	22 (5.7)	12 (3.2)	0.483	0.104
6C	19 (5.2)	16 (5.0)	7 (2.3)	22 (5.2)	11 (2.8)	13 (3.3)	15 (4.0)	0.356	0.238
15A	10 (2.7)	9 (2.8)	8 (2.6)	16 (3.8)	15 (3.9)	15 (3.9)	8 (2.2)	0.194	0.749
35F	5 (1.4)	3 (0.9)	6 (2.0)	14 (3.3)	18 (4.6)	9 (2.3)	4 (1.1)	**0.002**	0.301
31	15 (4.1)	7 (2.2)	14 (4.6)	12 (2.8)	6 (1.5)	14 (3.6)	9 (2.4)	0.424	0.269
16F	7 (1.9)	10 (3.1)	3 (1.0)	20 (4.7)	12 (3.1)	12 (3.1)	1 (0.3)	**0.008**	0.491
NT	9 (2.4)	8 (2.5)	10 (3.3)	3 (0.7)	8 (2.1)	7 (1.8)	9 (2.4)	0.727	0.538
21	3 (0.8)	6 (1.9)	7 (2.3)	8 (1.9)	11 (2.8)	5 (1.3)	4 (1.1)	0.064	0.939
34	3 (0.8)	8 (2.5)	5 (1.6)	8 (1.9)	7 (1.8)	4 (1.0)	4 (1.1)	0.377	0.596
37	1 (0.3)	4 (1.3)	1 (0.3)	3 (0.7)	4 (1.0)	6 (1.5)	2 (0.5)	0.518	0.373
24F	4 (1.1)	2 (0.6)	6 (2.0)	10 (2.4)	0 (0.0)	5 (1.3)	5 (1.3)	**0.044**	0.993
25A/38	7 (1.9)	2 (0.6)	0 (0.0)	0 (0.0)	1 (0.3)	1 (0.3)	3 (0.8)	0.252	0.118
13	2 (0.5)	3 (0.9)	1 (0.3)	1 (0.2)	2 (0.5)	1 (0.3)	2 (0.5)	0.966	0.552
7C	2 (0.5)	2 (0.6)	6 (2.0)	1 (0.2)	2 (0.5)	0 (0.0)	2 (0.5)	0.969	0.223
6D	0 (0.0)	0 (0.0)	0 (0.0)	0 (0.0)	0 (0.0)	3 (0.8)	0 (0.0)	0.973	0.096
35A	2 (0.5)	3 (0.9)	4 (1.3)	2 (0.5)	1 (0.3)	0 (0.0)	2 (0.5)	0.457	0.167
Other	6 (1.6)	8 (2.5)	8 (2.6)	5 (1.2)	4 (1.0)	7 (1.8)	7 (1.9)	-	

^a^Only serotypes detected in ≥3 isolates in at least 1 year are shown. The remaining serotypes are included in “Other” and include serotypes 18A, 1, 12B, 10B, 47F, 11F, 17A, 10F, 12F, 36, 11B, 7B, 11D, 35C, 18F, 42, 11C, 28A and 19B. PCV13, serotypes included in PCV13; addPCV15, the additional serotypes included in PCV15 which are not present in PCV13; addPCV20, the additional serotypes included in PCV20 which are not present in PCV15; addPPV23, the serotypes exclusively found in PPV23; NVT, non-vaccine serotypes. ^b^CA, Cochran Armitage test for trend for each of the indicated periods: 2016–2018 and 2012–2018. Values < 0.05 are indicated in bold.

In order to determine if some serotypes were more frequently associated with aIPD or aNIPP, differences in serotype distribution were evaluated for isolates responsible for aIPD (7) and aNIPP, between 2016 and 2018. Serotypes 4, 7F, 8, 12F, 14, 19A, 20, and 22F were associated with aIPD (all significant after FDR correction), while serotypes 10A, 11A, 19F, 21, 23A, 23B, 29/35B, 37, and NT isolates were associated with aNIPP (all significant after FDR correction). Serotypes 35F and 6C were also associated with aNIPP (*p* = 0.040 and *p* = 0.027 respectively, but unsupported after FDR correction).

### Antimicrobial susceptibility

The results of antimicrobial susceptibility testing, stratified by age group, are summarized in Table 4. A total of 211 isolates (18.4%) were classified as penicillin non-susceptible (PNSP), of which the majority expressed low-level resistance (97.6%, *n* = 206) and only 5 isolates (0.4%) expressed high-level resistance. According to current CLSI guidelines for parenteral penicillin in non-meningitis cases, only one isolate would have been considered PNSP, expressing intermediate resistance. A total of 243 isolates (21.1%) were found to be erythromycin resistant (ERP), of which 204 (84%) expressed the MLS_B_ phenotype and the remaining 39 (16%) presented the M phenotype. Simultaneous erythromycin resistance and PNSP (EPNSP) was found in 11.9% of the isolates (*n* = 137). During the current study period, there were no significant changes in antimicrobial resistance rates, except for penicillin, for which non-susceptibility increased from 17.0% in 2016 to 23.7% in 2018 (*p* = 0.018). Considering 2012–2018, resistance to tetracycline was found to have a decreasing trend (*p* = 0.014), but quite irregularly with significant yearly variations.

**Table 4:** Antimicrobial resistance of the isolates responsible for non-invasive pneumococcal pneumonia in adult patients in Portugal, 2016–2018.

	**No. of resistant isolates (%)** ^**a**^		
	**18–49 years (*****n** =* **246)**	**50–64 years (*****n** =* **322)**	≥**65 years (*****n** =* **581)**
PEN	44 (17.9)	54 (16.8)	113 (19.4)
${\text{MIC}}_{90}^{\text{b}}$	0.38	0.25	0.38
${\text{MIC}}_{50}^{\text{b}}$	0.012	0.012	0.016
CTX	3 (1.2)	1 (0.3)	7 (1.2)
${\text{MIC}}_{90}^{\text{b}}$	0.25	0.19	0.38
${\text{MIC}}_{50}^{\text{b}}$	0.016	0.016	0.016
LEV	1 (0.4)	1 (0.3)	9 (1.5)
ERY	60 (24.4)	69 (21.4)	114 (19.6)
CLI	52 (21.1)	60 (18.6)	92 (15.8)
CHL	1 (0.4)	4 (1.2)	7 (1.2)
SXT	45 (18.3)	42 (13.0)	93 (16.0)
TET	53 (21.5)	65 (20.2)	104 (17.9)

^a^PEN, penicillin; CTX, cefotaxime; LEV, levofloxacin; ERY, erythromycin; CLI, clindamycin; CHL, chloramphenicol; SXT, trimethoprim/sulfamethoxazole; TET, tetracycline. All isolates were susceptible to vancomycin and linezolid. ^b^MIC values are indicated in μg/ml. The MIC distribution was as follows: ≤ 0.064 μg/ml (n = 957), < 2 μg/ml (n = 187), 2 μg/ml (n = 4), 3 μg/ml (n = 1).

PCV7 serotypes accounted for 18.9% of PNSP, 33.0% of ERP and 38.7% of EPNSP while PCV13 serotypes accounted for 24.3, 37.7, and 52.6% for PNSP, ERP and EPNSP, respectively (Figure 1). The addPPV23 accounted for 44.6% of PNSP, 25.5% of ERP but only 4.4% of EPNSP, while NVT isolates accounted for 31.1% of PNSP, 36.8% of ERP and 43.1% of EPNSP. Serotype 19F was the most frequent serotype among ERP isolates, followed by serotypes 6C, 33F, and 11A, by decreasing order of frequency. PNSP isolates belonged mostly to serotypes 11A, 23B, and 14, while EPNSP isolates expressed serotypes 19F, 15A, 19A, and 14 most frequently (Figures 1–3).

## Discussion

The most notable feature of the 2016–2018 period is a stabilization of the serotype distribution causing aNIPP in Portugal. Results from previous studies documented a decline of PCV13 serotypes in aNIPP in the post-PCV13 era, mostly in the first years after the introduction of PCV13 in the private market (5, 19). However, in 2016–2018, during 3 years of higher infant PCV13 uptake following its introduction in the national immunization program, the PCV13 serotypes in aNIPP remained stable, with no change in serotype diversity when comparing to the pre-PCV13 period. When expanding this analysis to the period 2012–2018, a small decrease was seen in three of the additional serotypes present in PCV13 and not in PCV7, but not in all six addPCV13 serotypes.

When comparing with the serotype dynamics and distribution in aIPD in the same time period (7), the serotype changes were much less pronounced in aNIPP, but despite the differences there were also similarities. When considering 2012–2018 the increase in serotype 8 (although not statistically supported) was mirroring increases of this serotype in aIPD, which rose to become the dominant serotype in aIPD (7). The comparison between contemporary aIPD and aNIPP isolates identified mostly the same serotypes as before associated with each disease presentation (5), indicating that these are robust associations not affected by temporal serotype dynamics.

Serotype 3 was the leading cause of aNIPP, not associated with any age group, in contrast to aIPD where it was increasingly found with age (7). Serotype 3 is a major serotype in multiple disease presentations worldwide (7, 12, 15, 25–29). The persistence of this serotype as a cause of aIPD and aNIPP contrasts with the marked decreases in other PCV13 serotypes, possibly due to the herd effects of children vaccination, and may have multiple causes. A persistence of serotype 3 as a cause of all pneumococcal pneumonia (including bacteremic and aNIPP episodes) was also seen in recent studies from the UK, Spain and Canada (12, 30–32), indicating this may be a shared characteristic of the post-PCV13 epidemiology of pneumococcal pneumonia in various regions.

Serotype 11A, ranked second in aNIPP in 2016–2018, as in the previous study period (5). The considerable proportion of PNSP, ERP or EPNSP isolates expressing this serotype could partially explain its prevalence as a cause of pneumococcal disease, but isolates expressing this serotype are more rarely found in aIPD (5). This suggests there may be specific features of serotype 11A isolates that could make them particularly prone to cause aNIPP or be less virulent. Some of these isolates could represent the recently described clone present in southern Europe and causing IPD resulting from multi-fragment recombination events (33). Although serotype 11A isolates were also important causes of all pneumococcal pneumonia in one study in Spain (30), they were not particularly notable in another (32) nor in Canada (31) or in the UK, although serotype 11A increased in prevalence in the last study year in the UK study (12).

Despite almost two decades of use of PCVs targeting the PCV7 serotypes, the serotypes included in the first conjugate vaccine introduced in Portugal are still important causes of disease and are associated with high rates of antimicrobial non-susceptibility. Serotype 19F was the most frequent PCV7 serotype found among our collection, representing 6.1% of all aNIPP cases in 2016–2018, mostly associated with younger adults. Although the reasons behind its persistence are unclear, isolates expressing serotype 19F were associated with antimicrobial non-susceptibility, which, similarly to serotype 11A, could partly explain their resilience despite vaccine pressure. Other important PCV serotypes in aNIPP included 14 (PCV7) and 19A (addPCV13), which also remained approximately constant throughout the study period, although at a lower level than serotype 19F. As with serotype 19F, isolates expressing these serotypes were associated with antimicrobial non-susceptibility. Serotypes 14 and 19F were infrequent causes of pneumococcal pneumonia, if they were found at all, in Spain, UK and Canada, while serotype 19A tended to be more prevalent (12, 30–32), indicating there is also some national heterogeneity in the serotypes causing aNIPP, particularly among the PCV13 serotypes. In fact a recent meta-analysis identified serotype 19A as one of the most prevalent in NIPP (34) which was not the case in our study.

The stability of the proportion of aNIPP caused by PCV13 serotypes, in a situation of high vaccination coverage of children, suggests the herd effects and the modest use of the currently available vaccines for adults (PCV13 and PPV23) in Portugal are still insufficient to prevent a significant fraction of aNIPP. Moreover, antimicrobial resistance rates did not decrease, and we even found a significant increase in PNSP, which could be partially attributed to the persistence of vaccine serotypes such as 14, 19A, and 19F. When comparing antimicrobial resistance trends, differences between aIPD and aNIPP isolates were again evident, with the decrease in erythromycin and clindamycin resistance seen in aIPD (7) not being paralleled in aNIPP, which could be due to a larger number of serotypes associated with resistance in aNIPP, including serotypes 11A, 19F, and 23B, and, to a lesser extent, 14 and 19A.

All leading NVTs, except for serotype 9N, were significantly more frequent in aNIPP than in aIPD. Besides serotype 19F, all other serotypes associated with aNIPP were non-PCV13 serotypes, none were included in PCV15, and only serotypes 10A and 11A were included in PCV20. Despite the potential benefits of the use of higher valency PCVs in adults for the prevention of aNIPP, the existence of serotypes associated with aNIPP which are currently not covered by any PCV is worrying and justifies the continued monitoring of these infections to guide future vaccine formulations targeting these infections. This is illustrated by the fact that adding only four NVTs (23A, 23B, 9N, and 29/35B) to current formulations could potentially cover an additional 20% of aNIPP.

Our work has limitations, including the possibility that some of the isolates identified as being responsible for NIPP were in fact causing invasive disease (bacteremic pneumonia) or reflected colonization and not disease. Although there is a general recommendation for both blood and respiratory tract samples to be collected for the etiologic diagnosis of pneumonia, we cannot guarantee that this occurred in all cases. However, we believe that this did not introduce a significant bias, because if so, these would account for, at most, a small fraction of the isolates. Moreover, differences in serotype distribution found in this study for aIPD and aNIPP, strongly argues against this possibility. Since this is a laboratory-based study, it was not designed to collect information important to assess the severity of the infections caused by the different serotypes (e.g., hospitalization, ICU admission, 30-day mortality). The criteria for the presumptive diagnosis of pneumonia that triggered the collection of respiratory tract samples for microbiological testing may have also differed between and even within sites, since these were left to the attending physician and no guidelines were provided within the study. However, this does not compromise our approach of comparing the serotype distribution of aIPD and aNIPP cases. Importantly, the laboratories participating in both studies are the same and, although the populations served by each hospital are possibly different, the participating hospitals as a whole represent the diversity of the Portuguese population.

In the period immediately after the introduction of PCV13 in the national immunization plan for children with almost universal uptake, the serotype distribution of aNIPP remained approximately constant. NVTs account for a substantial fraction of aNIPP and continued surveillance may assist in identifying which serotypes may be more important to include in potential future vaccine formulations. The sustained persistence of serotype 3 is of special concern, because it is still a leading cause of pneumococcal infection in Portugal in both aIPD and aNIPP, even in a situation of near universal PCV13 use in children. The continued importance in aNIPP of several other PCV13 serotypes and of the serotypes included in PPV23 or in the higher valency PCVs currently entering the market, reinforces the potential benefits of increasing the use of vaccines to prevent pneumococcal disease in this population.

## Data availability statement

The original contributions presented in the study are included in the article/Supplementary material, further inquiries can be directed to the corresponding author.

## Author contributions

CS-C: Conceptualization, Data curation, Formal analysis, Investigation, Supervision, Validation, Writing – original draft, Writing – review & editing. JG-S: Data curation, Investigation, Writing – review & editing. AS: Formal analysis, Writing – review & editing. MR: Conceptualization, Data curation, Formal analysis, Project administration, Supervision, Validation, Writing – original draft, Writing – review & editing. JM-C: Conceptualization, Funding acquisition, Project administration, Resources, Validation, Writing – original draft, Writing – review & editing.
