# Supplementary material for: Adult non-invasive pneumococcal pneumonia in Portugal is dominated by serotype 3 and non-PCV13 serotypes 3-years after near universal PCV13 use in children
Source: Front Public Health. 2023 Dec 20;11:1279656. doi: 10.3389/fpubh.2023.1279656 (PMC10770798; doi:10.3389/fpubh.2023.1279656)
Supplement: Supplementary file 1 [file Data_Sheet_1.PDF]

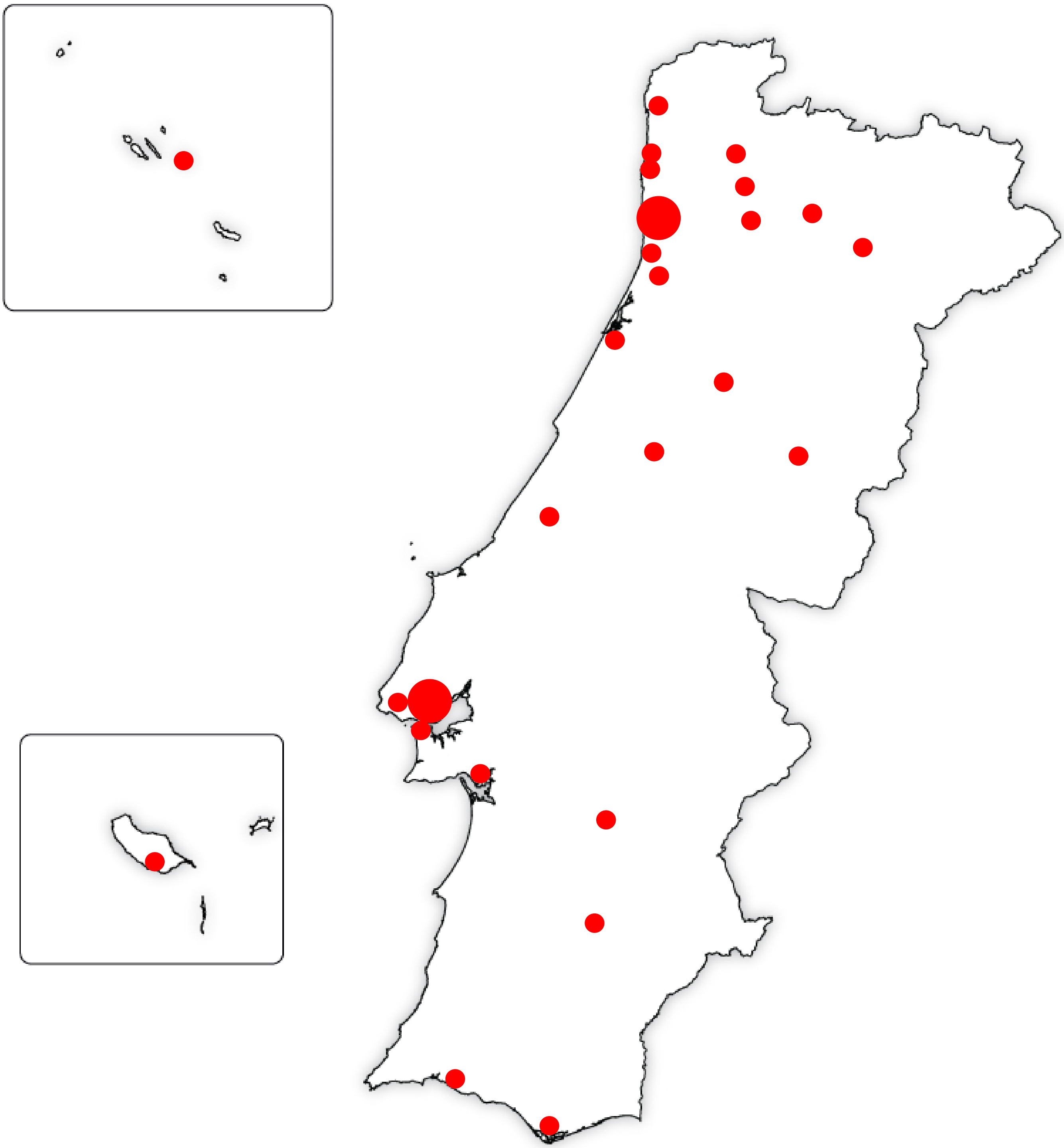

Figure S1 – Location in continental Portugal and the islands of Azores and Madeira of the participating laboratories. Each hospital is represented by a read dot. The larger dots in the metropolitan regions of Lisbon and Porto represent several hospitals.
